# Reliability and validity of the Mini-Eating and Drinking Ability Classification System (Mini-EDACS) among Dutch preschoolers with cerebral palsy

**DOI:** 10.1177/18758894251330469

**Published:** 2025-04-01

**Authors:** Floor van der Klift, Lynn B Orriëns, Bea Spek, Diane Sellers, Corrie E Erasmus, Karen van Hulst

**Affiliations:** 1 Merem Medical Rehabilitation, Almere, The Netherlands; 2 Department of Paediatric Neurology, Donders Institute for Brain, Cognition and Behaviour, Radboud University Medical Center, Amalia Children's Hospital, Nijmegen, The Netherlands; 3 Amsterdam UMC|University of Amsterdam, Department of Epidemiology and Data Science, Amsterdam, The Netherlands; 4 Chailey Clinical Services, Sussex Community NHS Foundation Trust, Brighton, UK; 5 Department of Rehabilitation, Donders Institute for Brain, Cognition and Behaviour, Radboud University Medical Center, Amalia Children's Hospital, Nijmegen, the Netherlands

**Keywords:** cerebral palsy, functional classification, eating/drinking, dysphagia, preschool-aged

## Abstract

**Purpose:**

This study aimed to translate the English version of the Mini-Eating and Drinking Ability Classification System (Mini-EDACS) into Dutch and assess its psychometric properties and applicability among preschool-aged children with cerebral palsy (CP) in the Netherlands.

**Methods:**

Forty-eight children with CP (18–36 months) were included. Inter-rater reliability of the Dutch version of the Mini-EDACS was assessed between two speech and language therapists (SLTs) and between two SLTs and parents. Construct validity was established by hypothesis testing regarding the expected strength of the correlation between Mini-EDACS level and sum score of (a) the Pediatric Eating Assessment Tool (PEDI-EAT-10) and (b) the Montreal Children's Hospital Feeding Scale (MCH-FS).

**Results:**

The level of agreement for Mini-EDACS level was almost perfect between SLTs (weighted kappa (k_w_) = 0.83) and substantial between parents and SLTs (parents vs SLT-1: k_w _= 0.77; parents vs SLT-2: k_w _= 0.70).

Kendall's tau-b correlation between Mini-EDACS and PEDI-EAT-10 was 0.66 (p < 0.001), slightly lower than hypothesized, and 0.52 (p < 0.001) between Mini-EDACS and MCH-FS, aligning with the hypothesis. Applicability was found to be good.

**Conclusion:**

The Dutch version of the Mini-EDACS showed sufficient inter-rater reliability, construct validity and applicability and can be used in clinical care in the Netherlands to promote unambiguous communication between healthcare professionals and parents.

## Introduction and rationale

Cerebral palsy (CP) is described as a group of neurological disorders attributed to non-progressive damage or abnormalities in the developing foetal or infant brain. It has a worldwide prevalence of 2-3 per 1000 live births^1^ and is often accompanied by problems in motor skills, sensation, perception, cognition, communication and behaviour.^2^ In addition, children with CP are at an increased risk of dysphagia and feeding problems compared to typically developing children.^3^ The prevalence of dysphagia in children with CP is estimated between 27% and 99% and has been shown to increase with the severity of CP.^4^ An estimated 39% of children with CP below the age of three need food texture modifications and 10% are dependent on tube feeding.^5^

Children with dysphagia frequently experience problems such as malnutrition, dehydration and limited growth. Moreover, pulmonary infections often occur because of aspiration (i.e., particles of food, fluid, stomach contents or saliva entering the lungs), which is associated with an increased mortality risk.^3,6^ Early intervention for dysphagia and feeding difficulties can prevent serious respiratory disease and hospitalizations.^7^ In addition to health risks, dysphagia and feeding problems often lead to tension at mealtimes, impacting not only the child but also their family and caregivers.^3^ This stress can alter behaviour and disrupt interactions between parents and children.^8^

In rehabilitation care, various classification systems are used to assess the functional abilities of children with CP, including mobility, communication, speech, and eating and drinking abilities.^9–20^ Focusing on functional capacity, these classification systems facilitate unambiguous communication among healthcare professionals and can bridge clinical care with research findings.^21,22^ In addition, collaborating with parents in the classification process enhances shared decision-making.^23^ The Eating and Drinking Ability Classification System (EDACS) was developed to classify the eating and drinking abilities of children and young people (aged three to 21 years) with CP in daily life. The EDACS standardizes the categorization of these abilities, aiding in assessment of patients, communication about risks, and planning of interventions for individuals facing challenges in these areas. It classifies individuals’ usual eating and drinking abilities into one of five levels and assesses required assistance on a separate three-level scale.^19,20^ Recently, the Mini-EDACS was developed to supplement the original EDACS for use with infants from the age of 18 months to 36 months old,^24^ who previously fell below the age range of EDACS. The aim of the current study was to translate the Mini-EDACS into Dutch and assess its psychometric properties and applicability for children with CP aged 18–36 months.

## Methods

**Figure 1.**
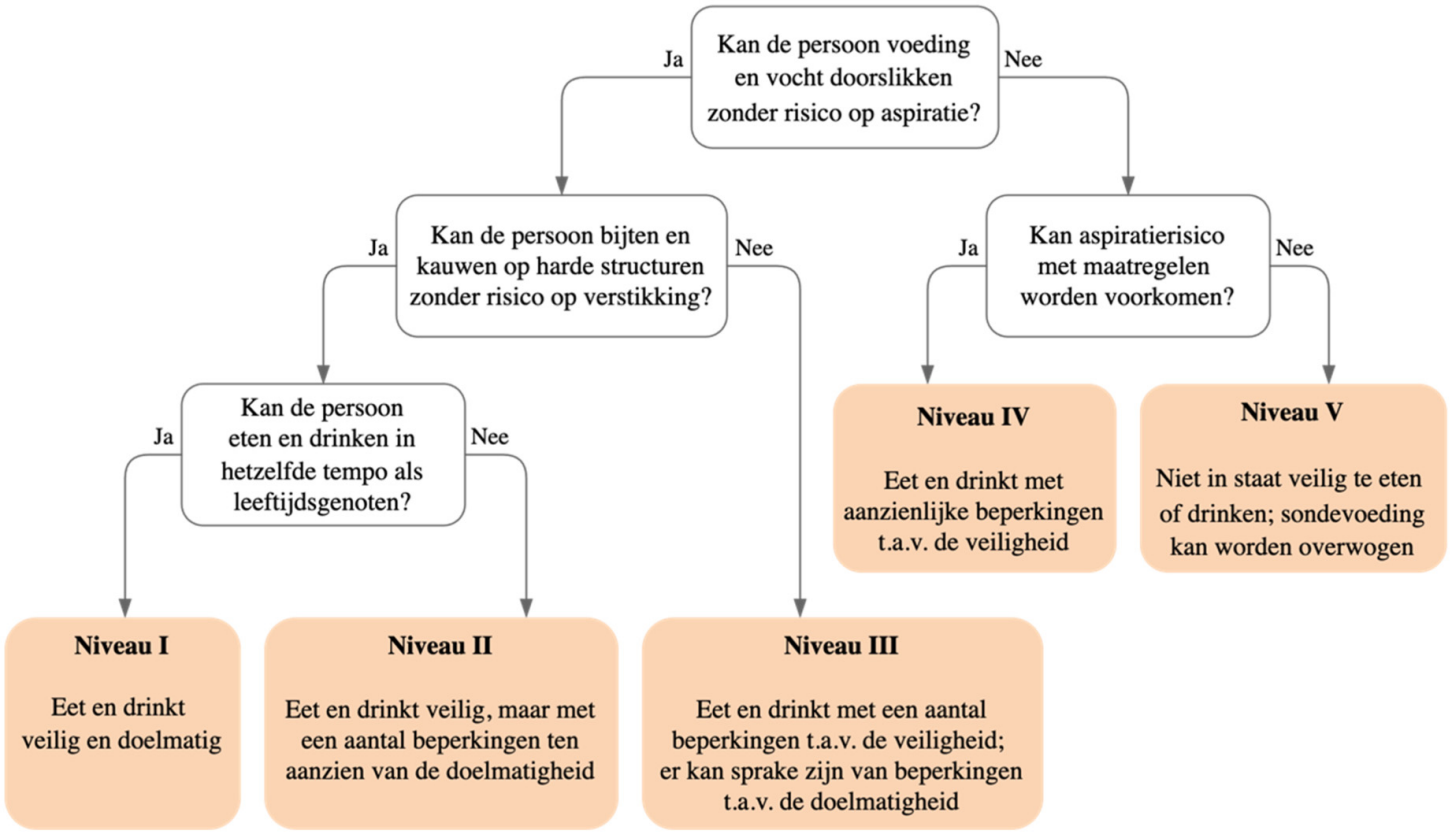
Clinical algorithm Mini-EDACS in Dutch. Mini-EDACS: Mini-Eating and Drinking Ability Classification System.

This cross-sectional study consisted of two phases. In the first phase, the Mini-EDACS’ clinical algorithm (Figure 1) and manual were translated into Dutch by two members of the research group using the FACIT translation methodology,^25^ ensuring both translation and bridging of cross-cultural differences. In the second phase, inter-rater reliability, construct validity, and applicability of the Mini-EDACS were assessed. Data collection for this phase occurred from November 2021 to June 2023.

### Setting and participants

Speech and language therapists (SLTs) working in paediatric rehabilitation centres, outpatient clinics for children born prematurely and primary care throughout the Netherlands were approached to participate in the study. These SLTs and their respective colleagues (e.g., occupational therapists and rehabilitation physicians) shared study information with parents/caregivers (hereafter referred to as parents) to recruit children for the study. Children between the ages of 18 and 36 months with a diagnosis or strong suspicion of CP as indicated by a paediatric neurologist or paediatrician were included. Children were excluded if they were suffering from a temporary illness that could affect swallowing (e.g., cold or flu) during study procedures or if parents could not understand the instructions of the Mini-EDACS in Dutch. The aim was to include 50 children, as this sample size is recommended for the accurate assessment of the reliability and validity of an outcome measure.^26^ After parents signed informed consent, their children were included in the study.

### Measurements

Construct validity was assessed by testing *a priori* hypotheses, specifying the expected direction and magnitude of the correlation. This approach is in accordance with the COnsensus-based Standards for the selection of health Measurement INstruments (COSMIN) guidelines, developed by international researchers with expertise in the development and evaluation of outcome measures to provide standards for good methodological quality.^27^ The correlation between the Mini-EDACS level and two parent-reported questionnaires, the Dutch version of the Montreal Children's Hospital Feeding Scale (MCH-FS)^8,28^ and the Pediatric Eating Assessment Tool (PEDI-EAT-10),^29^ which are designed to measure similar constructs, were evaluated. For the purpose of this study, the PEDI-EAT-10 was translated into Dutch, with the permission from the original author. As the Mini-EDACS and the PEDI-EAT-10 both describe safety and efficiency of eating and drinking, with overlapping topics, at least a moderate to strong positive correlation (τ ≥ 0.70) was hypothesized. The MCH-FS, however, questions a broader area of feeding and nutritional problems than the Mini-EDACS and includes questions about behaviour and stress around mealtime and so a moderate positive correlation (τ= 0.4-0.6) between the MCH-FS sum score (after inversion of items 1, 3, 4, 8, 10, 12, and 13) and Mini-EDACS level was hypothesized.

### Mini-EDACS

The Mini-EDACS consists of a clinical algorithm leading towards a five-level ordinal scale (Figure 1). Level I represents ‘eats and drinks safely and efficiently’ and level V represents ‘unable to eat or drink safely; tube feeding may be considered to provide nutrition’. The scale is accompanied by detailed descriptions of the five levels and differences between the levels to aid in classification. A separate three-level ordinal scale is included to describe the child's level of assistance required at mealtimes: ‘independent’, ‘requires assistance’, and ‘totally dependent’. The sections outlining the Purpose, Background, Key Features, User Instructions, General Headings, and Definitions and Distinctions between the levels are consistent for both the EDACS and Mini-EDACS. However, the individual level descriptions for the Mini-EDACS (I-V) are adapted to this younger age group, creating a separate set of descriptors for individuals aged 18 to 36 months.^24^

### MCH-FS

The Dutch version of the MCH-FS (Screeningslijst Eetgedrag Peuters)^28^ is a parent-reported questionnaire that aims to quickly identify a child's feeding problems. The scale comprises 14 items, each evaluated using a seven-point Likert scale, resulting in a sum score (maximum 98) with a corresponding percentile score. A percentile score between p90-p95 indicates ‘mild problems’, whereas a percentile score > p95 indicates ‘moderate to severe problems’. The psychometric properties of the scale were studied in a group of parents (n = 1448) with typically developing children aged six months to four years in the Netherlands. Cronbach's alpha was adequate for both total score (0.84) and two subscales: negative mealtime behaviours (0.82) and negative causes and consequences (0.75), suggesting a robust internal consistency. Because the two factors of the subscales correlate strongly, the use of one factor (total MCH-FS score) is legitimized.

### PEDI-EAT-10

The PEDI-EAT-10^29^ is a parent-reported tool that aims to identify swallowing difficulties and their effects in a paediatric population. The tool consists of ten questions with a five-point Likert scale and results in a maximum sum score of 40. A sum score of four and above is interpreted as abnormal.^29^ The PEDI-EAT-10 was independently translated into Dutch by three of the authors and the translation was finalized through consensus. The PEDI-EAT-10 has been shown to be a reliable and valid instrument as studied in Turkey (but not yet in the Netherlands) in 138 children with spastic CP aged 18 months to 18 years.

### Procedures

#### Parents

Parents were asked to classify the safety and efficiency of their children's usual eating and drinking, as well as the level of assistance required, using the Mini-EDACS, for which they received a short version of the manual. Additionally, participants completed the Dutch MCH-FS (27), the PEDI-EAT-10 (28), and a questionnaire encompassing demographic and clinical characteristics of their child, including age, sex, motor skills, incidence of respiratory infections, source of nutrition (tube, oral, or combination), food textures, and other functional eating habits.

Parents were instructed to capture three to five-minute video recordings during three standard mealtimes (e.g., breakfast, lunch, snack, or dinner) spanning one or two consecutive days. These recordings were intended to encompass a variety of foods and beverages, ideally comprising solid, thick liquid, and thin liquid textures. Parents were prompted to present both foods and fluids that children were accustomed to but also to include challenging textures for their child.

The videos were recorded with the arQive Camera app and stored in a safe online location.^30^ Finally, four questions were asked regarding the applicability of the Mini-EDACS: “How clear were the descriptions of the Mini-EDACS levels?”, “How clear were the descriptions of the differences between the Mini-EDACS levels?”, “How difficult did you find classifying the Mini-EDACS?”, and “How much time did you spend classifying the Mini-EDACS?”.

#### SLTs

Participating SLTs took part in a one-and-a-half-hour virtual training session. This training was intended to familiarize them with the Mini-EDACS and explain potential issues that might arise in choosing between levels in this younger age group, in which children are learning new eating and drinking skills. In clinical practice, the majority of SLTs have experience and are trained in the use of the EDACS in older children. To reduce the likelihood that classifying SLTs were familiar with particular children, children were allocated based on considerable geographical separation from the SLTs.

Each SLT was assigned a cohort comprising three to five children for the purpose of categorizing both the Mini-EDACS level and the level of assistance required, all of which were determined from video recordings. Each participating child was classified independently by two of the participating SLTs. The SLTs were given access to more information about the clinical and demographic characteristics of the children but were blinded to the Mini-EDACS level, level of assistance required, and MCH-FS and PEDI-EAT-10 scores as classified by the parents. Finally, all SLTs responded to the same four questions as parents regarding the applicability of the Mini-EDACS.

### Statistical analysis

Patient characteristics as well as Mini-EDACS applicability ratings were summarized using the appropriate descriptive statistics.

Inter-rater reliability of the (1) Mini-EDACS levels and (2) level of assistance required were determined between parents and both classifying SLTs and between the two (classifying) SLTs using quadratic weighted kappa (k_w_). Weighted kappa was interpreted following Landis and Koch.^31^ In addition, a Wilcoxon signed ranks test was performed to test for overall systematic differences in ratings between parents and SLT-1 and parents and SLT-2.

To assess construct validity, correlations were calculated between the Dutch MCH-FS^28^ and PEDI-EAT-10^29^ with the Mini-EDACS level as classified by parents, using Kendall's tau-b (τ), which was interpreted following Cohen.^32^ P-values <0.05 were considered to be statistically significant. All statistical analyses were performed using SPSS version 27.

A sensitivity analysis was conducted to investigate the impact of utilizing adjusted ages rather than chronological ages for children born prematurely on the inter-rater reliability, construct validity, and applicability of the Mini-EDACS. Given that the Mini-EDACS is designed for children aged 18–36 months and premature children's ages are adjusted up to 24 months, it was possible that some included children had adjusted ages below 18 months. Consequently, analyses were reiterated, excluding children whose adjusted ages fell outside the designated eligibility age range.

## Results

### Participants

In total, 55 children with a diagnosis or strong suspicion of CP between the ages of 18–36 months were initially included. Seven children were ultimately excluded from the analysis due to incomplete submission of parental questionnaires or videos. Characteristics of the 48 participating children are shown in Table 1.

**Table 1.** Characteristics of included children.

Median age (months (IQR))	26.7 (9.0)
Age range (months)	18–36
Sex	
Male	21 (44)
Female	27 (56)
Speech and language therapy for feeding problems	
Yes	19 (40)
No	29 (60)
Feeding technique	
Oral feeding	44 (92)
Tube feeding	2 (4)
Oral and tube feeding	2 (4)
Premature birth (<37 weeks)	
Yes	18 (38)
No	30 (62)
Motor skills	
Walking	22 (46)
Walking with support	4 (8)
Not walking (yet)	22 (46)

Data are n (%) unless otherwise stated. IQR: interquartile range.

No major differences were found for the inter-rater reliability, construct validity, and applicability of the Mini-EDACS when excluding children (n = 2) based on adjusted age rather than chronological age. Therefore, the findings reported reflect the full cohort of 48 children.

Twenty-nine SLTs participated in classifying children using the Mini-EDACS. All had experience working with children with CP; one had 1–5 years of experience, five had 5–10 years of experience and 23 had more than 10 years of experience. Each child was classified independently by two of these SLTs, with each SLT classifying three to five children.

### Inter-rater reliability

The agreement on the Mini-EDACS level between SLTs was almost perfect (k_w_: 0.83; 95% CI: 0.73-0.92), as interpreted by Landis and Koch.^31^ This suggested a high level of consistency in the classifications. SLTs fully agreed in 28 out of 48 cases (58%). There was a one-level difference for 19 children, while for one child there was a two-level difference (Table 2). The agreement on Mini-EDACS levels between parents and SLTs was substantial (k_w_ for parents vs SLT-1: 0.77; 95% CI: 0.64-0.91; k_w_ for parents vs SLT-2: 0.70; 95% CI: 0.54-0.85) (Table 3). The agreement on the level of assistance required between SLTs was substantial (k_w_: 0.70; 95% CI: 0.50-0.90), with perfect agreement in 36 out of 48 children (75%). There was a one-level difference in 10 children and a two-level difference in two children (Table 4). The agreement on the level of assistance required between parents and SLTs was substantial (k_w_: 0.73; 95% CI: 0.54-0.91 for parents vs SLT-2) to almost perfect (k_w_: 0.81; 95% CI: 0.70-0.92 for parents vs SLT-1) (Table 5). A Wilcoxon signed-rank test indicated a statistically significant distinction between SLTs’ classification across the five Mini-EDACS levels compared to parental assessments: SLTs tended to indicate greater challenges pertaining to eating and drinking safety and efficiency in children than parents did (Z: −2.82, p = 0.01 for parents vs SLT-1; Z: −2.45, p = 0.02 for parents vs SLT-2). Moreover, SLTs appeared to rate the level of assistance required higher than parents, although not statistically significantly (Z: −1.73, p = 0.15 for parents vs SLT-1; Z: −1.89, p = 0.09 for parents vs SLT-2). However, these results should be interpreted with slight caution due to an increased risk of a type 1 error, considering the multitude of tests that were performed.

**Table 2.** Agreement in Mini-EDACS levels between SLT-1 and SLT-2.

		Mini-EDACS level by SLT-1					
		I	II	III	IV	V	Total
Mini-EDACS level by SLT-2	I	11	3	1	0	0	15
	II	9	6	3	0	0	18
	III	0	1	4	2	0	7
	IV	0	0	0	6	1	7
	V	0	0	0	0	1	1
Total		20	10	8	8	2	48

Shaded cells indicate absolute agreement between SLT-1 and SLT-2.

Abbreviations: Mini-EDACS: Mini-Eating and Drinking Ability Classification System; SLT: speech and language therapist.

**Mini-EDACS level:** Level I represents ‘eats and drinks safely and efficiently’, level II represents ‘eats and drinks safely but with some limitations to efficiency’, level III represents ‘eats and drinks with some limitations to safety; there may be limitations to efficiency’, level IV represents ‘eats and drinks with significant limitations to safety’, and level V represents ‘unable to eat or drink safely—tube feeding may be considered to provide nutrition’.

**Table 3.** Agreement in Mini-EDACS levels between parents and SLT-1.

		Mini-EDACS level by SLT-1					
		I	II	III	IV	V	Total
Mini-EDACS level by parents	I	18	2	4	0	0	24
	II	2	6	2	3	0	13
	III	0	0	3	3	0	6
	IV	0	0	1	2	0	3
	V	0	0	0	0	2	2
Total		20	8	10	8	2	48

Shaded cells indicate absolute agreement between parents and SLT-1.

Abbreviations: Mini-EDACS: Mini-Eating and Drinking Ability Classification System; SLT: speech and language therapist.

**Mini-EDACS level:** Level I represents ‘eats and drinks safely and efficiently’, level II represents ‘eats and drinks safely but with some limitations to efficiency’, level III represents ‘eats and drinks with some limitations to safety; there may be limitations to efficiency’, level IV represents ‘eats and drinks with significant limitations to safety’, and level V represents ‘unable to eat or drink safely—tube feeding may be considered to provide nutrition’.

**Table 4.** Agreement of level of assistance SLT-1 and SLT-2.

		level of assistance by SLT-1			Total
		I	II	III	
Level of assistance by SLT-2	I	16	5	0	21
	II	3	11	1	15
	III	2	1	9	12
Total		21	17	10	48

Shaded cells indicate absolute agreement between SLT-1 and SLT-2.

Abbreviations: SLT: speech and language therapist. **Level of assistance:** I: independent, II: requires assistance, III: totally dependent.

**Table 5.** Agreement of level of assistance between parents and SLT-1.

		level of assistance by SLT-1			Total
		I	II	III	
Level of assistance by parents	I	20	8	0	28
	II	1	7	1	9
	III	0	2	9	11
Total		21	17	10	48

Shaded cells indicate absolute agreement between parents and SLT-1.

Abbreviations: SLT: speech and language therapist. **Level of assistance:** I: independent, II: requires assistance, III: totally dependent.

### Construct validity

**Figure 2.**
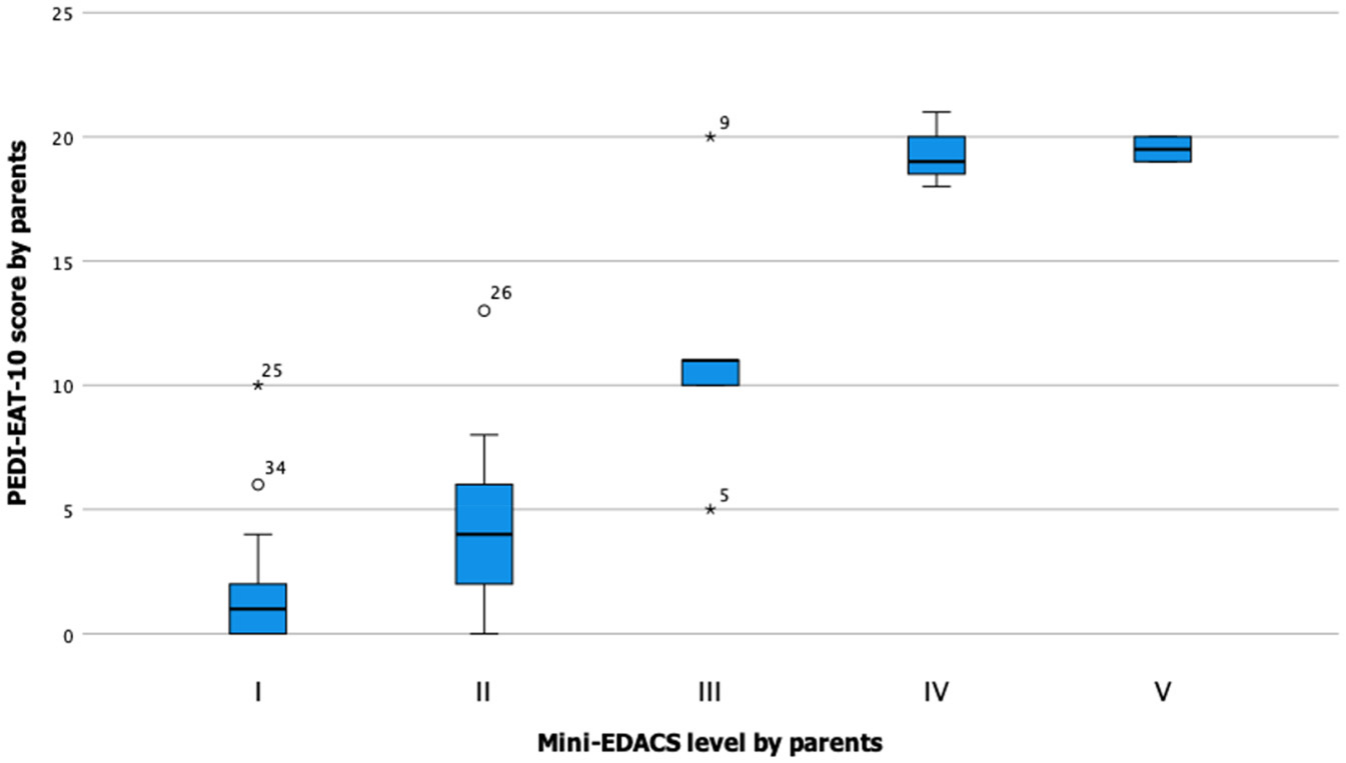
Box plots displaying the association between the PEDI-EAT-10 sum score and the Mini-EDACS level by parents.

Abbreviations: PEDI-EAT-10: Pediatric Eating Assessment Tool; Mini-EDACS: Mini-Eating and Drinking Ability Classification System.

There was a moderate positive correlation between Mini-EDACS levels and PEDI-EAT-10 sum scores (τ= 0.66, p < 0.001), which was slightly lower than the hypothesized τ = 0.7. More variation in PEDI-EAT-10 sum score was found in the lower Mini-EDACS levels than in the higher levels (Figure 2).

**Mini-EDACS level:** Level I represents ‘eats and drinks safely and efficiently’, level II represents ‘eats and drinks safely but with some limitations to efficiency’, level III represents ‘eats and drinks with some limitations to safety; there may be limitations to efficiency’, level IV represents ‘eats and drinks with significant limitations to safety’, and level V represents ‘unable to eat or drink safely—tube feeding may be considered to provide nutrition’.

**Figure 3.**
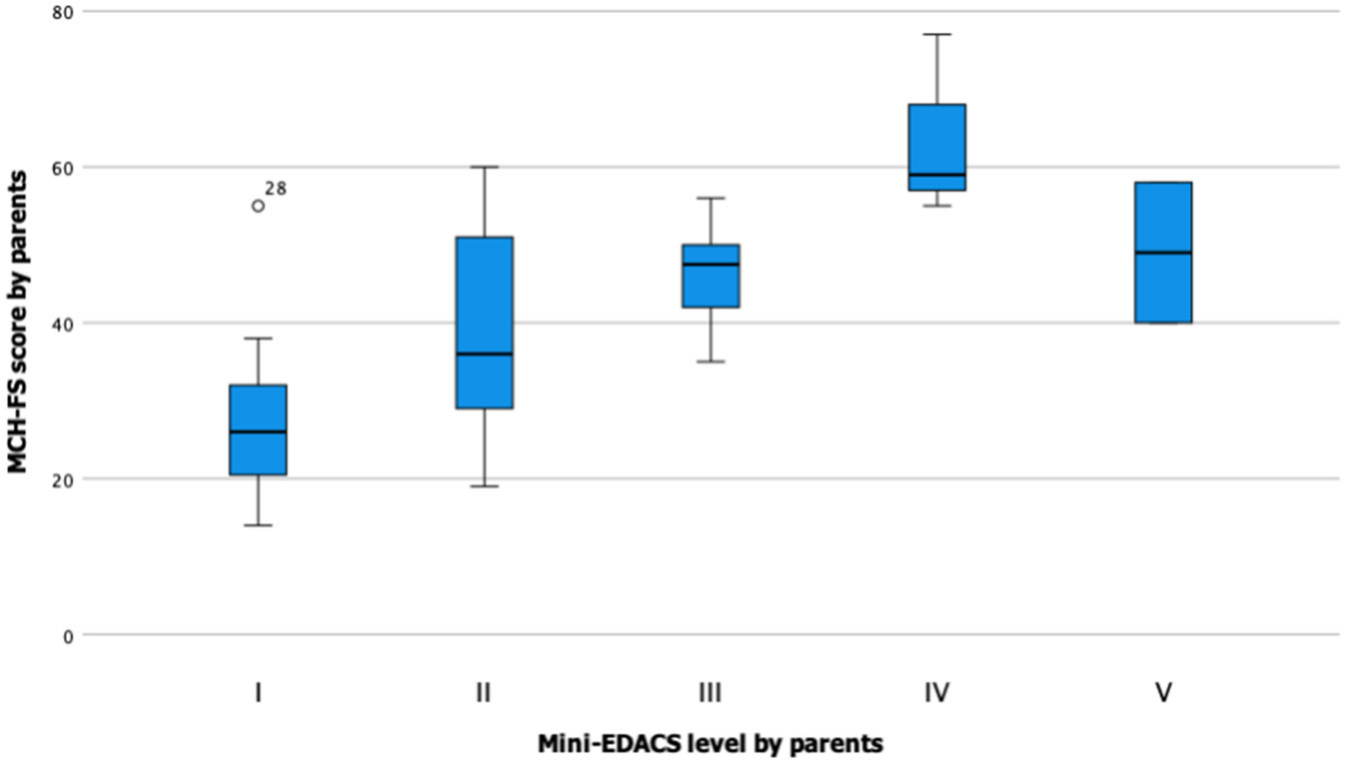
Box plots displaying the association between the MCH-FS sum score and the mini-EDACS level by parents.

Abbreviations: MCH-FS: Montreal Children's Hospital Feeding Scale; Mini-EDACS: Mini-Eating and Drinking Ability Classification System.

In accordance with the hypothesis, there was a moderate correlation between the Mini-EDACS level and MCH-FS sum score (τ = 0.52, p < 0.001). Again, more variation in MCH-FS sum score was found in the lower Mini-EDACS levels (Figure 3).

**Mini-EDACS level:** Level I represents ‘eats and drinks safely and efficiently’, level II represents ‘eats and drinks safely but with some limitations to efficiency’, level III represents ‘eats and drinks with some limitations to safety; there may be limitations to efficiency’, level IV represents ‘eats and drinks with significant limitations to safety’ and level V represents ‘unable to eat or drink safely—tube feeding may be considered to provide nutrition’.

### Applicability

**Figure 4.**
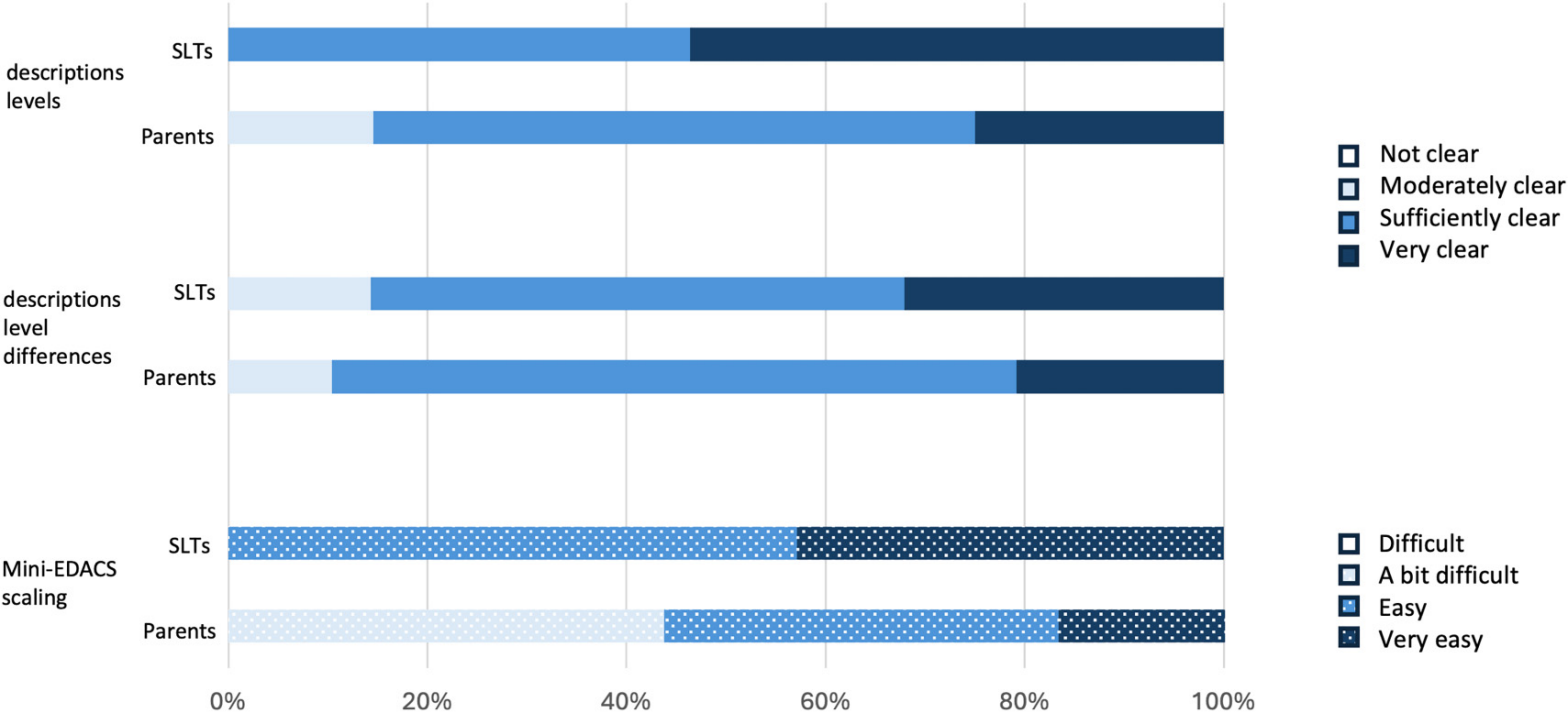
Applicability of the Mini-EDACS by SLTs and parents.

Abbreviations: Mini-EDACS: Mini-eating and drinking ability classification system; SLT: speech and language therapist.

The applicability of the Mini-EDACS was evaluated across four domains, both by SLTs and parents (Figure 4). All SLTs and most parents (85%) found the descriptions of the Mini-EDACS ‘sufficiently clear’ to ‘very clear’. Similarly, 86% of SLTs and 90% of parents found the descriptions of the differences between levels ‘sufficiently clear‘ to ‘very clear’. Although all SLTs rated the difficulty of scaling the Mini-EDACS as ‘easy’ or ‘very easy’, 44% of parents found it ‘a bit difficult’ to classify their child's eating and drinking abilities. SLTs spent an average of 17.6 min (range: 2–30 min) in classifying the Mini-EDACS, whereas parents spent an average of 8.3 min (range: 1–30 min).

**Question 1:** How clear were the descriptions of the Mini-EDACS levels?

**Question 2:** How clear were the descriptions of the differences between the Mini-EDACS levels?

**Question 3:** How difficult did you find it to scale the Mini-EDACS?

## Discussion

Previously, no classification system for assessing functional eating and drinking abilities in children with CP under the age of three was accessible for the Dutch population. In this study, the Dutch version of the Mini-EDACS was shown to be a reliable, valid, and applicable classification system. It facilitates clear and consistent communication among healthcare providers and parents about the child's individual eating and drinking abilities.

While inter-rater reliability was generally satisfactory, agreement on the Mini-EDACS level between two SLTs seemed to be greater than between parents and SLTs. This discrepancy is understandable, given that SLTs applied a consistent professional perspective while classifying these children from video. Notably, SLTs tended to assign children to a higher Mini-EDACS level compared to parents, potentially influenced by their awareness of associated risks in eating and drinking behaviours. This aligns with findings from the original EDACS study.^20^ This difference in perspective is consistent with findings from studies of the Communication Function Classification System^15^ and Gross Motor Function Classification System.^33^ Parents tended to rate their children's abilities, both in communication and motor function, as more effective or more limited than professionals, who observed children in more structured settings. This discrepancy is likely due to parents’ broader exposure to their child's daily performance in varied environments, while professionals focus on specific clinical risks, such as ‘safety and efficiency’ related to eating and drinking in this study.

Agreement on the level of assistance required among SLTs was slightly lower than that for Mini-EDACS levels. This could be due to the inherent dependence of children in this age group, making it challenging to differentiate between pathological and normal dependency during eating and drinking.

It was observed that SLTs were less likely to agree on the lowest Mini-EDACS levels, showing greatest functional ability, which resulted in frequent disagreement of one level. It is possible that SLTs have less experience observing the eating and drinking abilities of typically developing children, adopting a more risk averse viewpoint when looking at eating and drinking of young developing children. Typically developing children of a young age show a wide variability in eating skills^34^ and may show signs of aspiration like coughing and wet breath, irrespective of whether they are diagnosed with CP.^35^

In contrast to the previous Dutch EDACS study,^20^ the current study revealed a few cases where disagreements on Mini-EDACS level extended beyond a single level. On the one hand, this may underscore the potential challenge parents face in distinguishing between normal and abnormal eating and drinking abilities, especially given the diverse range in eating abilities in young children with CP.^34^ On the other hand, parents may have unique insights into their child's abilities that may not be fully discernible through the SLTs’ observations. The authors propose that the Mini-EDACS is a valuable tool to foster dialogue with parents about their child's functional eating and drinking capacity. Using the Mini-EDACS level classified by parents as a starting point could provide insights into potential differences in perspective between parents and SLTs.

Regarding construct validity, correlations between Mini-EDACS and PEDI-EAT-10, as well as Mini-EDACS and MCH-FS, supported the study hypotheses. Interestingly, the correlation between Mini-EDACS level and MCH-FS sum score appeared stronger at higher Mini-EDACS levels but diverged at lower levels. This discrepancy may stem from MCH-FS's broader assessment scope, which includes psychosocial aspects beyond safety and efficiency (8). For example, a child may face issues like selective eating or behavioural problems, leading to an elevated MCH-FS sum score even if safety and efficiency of eating are not compromised, potentially resulting in a lower Mini-EDACS level.

Affirming applicability, both SLTs and parents found the descriptions of the Mini-EDACS levels and distinctions between them as ‘moderately clear’ to ‘very clear’. However, the time taken to classify the Mini-EDACS varied substantially between SLTs and parents. This difference is explicable because parents did not need to watch videos or review additional information about the children, unlike SLTs.

### Clinical implications

The availability of the Mini-EDACS in the Netherlands offers valuable advantages, facilitating prompt referral to early treatment groups, potentially preventing secondary complications, and enhancing parent well-being. In line with recommendations by Gibson et al., classification of eating and drinking ability under the age of three is crucial for preventing and managing respiratory problems in young children with CP.^36^

Moreover, collaborative classification with parents can expand parents’ knowledge and therefore contributes to shared decision-making about daily care and potential interventions.^23^ Additionally, the Mini-EDACS could facilitate dialogue between SLTs and parents regarding shared and realistic goal setting.^37,38^ In the context of shared decision-making, acknowledging and valuing parents’ expertise concerning their own child is of great importance.^39^

Importantly, extra caution is required when using a classification system for young children with CP, considering that parents may still be navigating an acceptance process. It is important to emphasize that a Mini-EDACS classification is not an additional definitive diagnosis but functions as a monitoring system to evaluate the safety and efficiency of eating and drinking. As advocated by Scime et al., acknowledging parents in their acceptance process is essential, along with recognizing the limitations of the classification system and placing it within a broader and holistic context.^40^

Stability of the Mini-EDACS classification over time should be investigated, especially considering the finding that Gross Motor Function Classification System levels in infants are less precise than in older children.^41^ Considering the potential for development and learning at a young age, it is likely that more frequent reclassification is required compared to older children with CP.^42^ Therefore, until the stability of the Mini-EDACS has been studied, the recommendation is to reclassify the Mini-EDACS during an annual evaluation.

It is notable that classification systems, such as the Mini-EDACS, typically do not stipulate whether chronological age or adjusted age should be used. Given the common occurrence of prematurity among children with CP, necessitating age adjustment until 24 months,^41^ defining the age parameters of the classification system appears to be important. Further exploration of differences in the applicability of the Mini-EDACS, through a subgroup analysis on a larger sample, specifically within the age group of 18–24 months, could provide valuable insights.

### Strengths and limitations

Strengths of this study included the use of videos of mealtimes to classify eating abilities in daily life (i.e., classification based on a realistic display of safety and efficiency) and the provision of demographic and clinical patient characteristics to help SLTs in classifying the Mini-EDACS, both contributing to the external validity of the findings.^24^ In addition, two SLTs scored the child's Mini-EDACS level based on videos of the exact same mealtimes, which eliminated the possibility of differences in observations and contributed to an efficient assessment of reliability. However, there were also some limitations that should be considered. The sample size of 48 children fell slightly short of the recommended 50 for a validity study, although it is still considerable for this age group.^26^ Furthermore, there appeared to be a general homogeneity in Mini-EDACS levels within the current sample, with a predominance of children classified in the lower levels, which may have increased the difficulty of reaching sufficient inter-rater reliability.^26^ While video-based classification facilitates standardized assessments, it also introduces some constraints, including lack of control over the duration of the observation, the child's posture, or the type of food. Moreover, relevant contextual information (e.g., knowledge about performance in other situations) was unavailable during the current study, which could have negatively affected the accuracy of classification. Although the PEDI-EAT-10 has been validated in other countries, it has not yet been validated in Dutch population, which may be considered as a limitation of this study. Therefore, construct validity based on the PEDI-EAT-10 should be interpreted with appropriate caution. The need to validate the PEDI-EAT-10 in the Netherlands has become evident and members of the study team will focus on this in 2025. Despite these limitations, this study identified substantial to almost perfect reliability, which signals promising potential for the reliability of the Mini-EDACS.

## Conclusion

The Dutch version of the Mini-EDACS demonstrated adequate inter-rater reliability, construct validity and applicability, making it a valuable tool for daily practice with pre-schoolers with CP. However, it is important to consider that the reliability of the Mini-EDACS in assessing the level of assistance required appears to be lower than that for the overall assessment of eating and drinking abilities in this age group. To enhance its utility, future research should evaluate the stability of classifications over time to determine how frequently the Mini-EDACS level should be reassessed. In summary, the Mini-EDACS provides a comprehensive approach to evaluating and supporting the eating and drinking abilities of children with CP, contributing to safer, more effective, and personalized care strategies.
